# Phenotypic and genotypic antimicrobial resistance in *Escherichia coli* strains isolated from household dogs in Chile

**DOI:** 10.3389/fvets.2023.1233127

**Published:** 2023-08-16

**Authors:** Nicolás Galarce, Gabriel Arriagada, Fernando Sánchez, Beatriz Escobar, Mauricio Miranda, Sofía Matus, Rocío Vilches, Camila Varela, Carlos Zelaya, Josefa Peralta, Esteban Paredes-Osses, Gerardo González-Rocha, Lisette Lapierre

**Affiliations:** ^1^Escuela de Medicina Veterinaria, Facultad de Ciencias de la Vida, Universidad Andrés Bello, Santiago, Chile; ^2^Instituto de Ciencias Agroalimentarias, Animales y Ambientales, Universidad de O’Higgins, San Fernando, Chile; ^3^Programa de Doctorado en Ciencias Silvoagropecuarias y Veterinarias, Universidad de Chile, Santiago, Chile; ^4^Departamento de Medicina Preventiva Animal, Facultad de Ciencias Veterinarias y Pecuarias, Universidad de Chile, Santiago, Chile; ^5^Departamento de Salud Ambiental, Instituto de Salud Pública de Chile, Santiago, Chile; ^6^Instituto de Ciencias Naturales, Facultad de Medicina Veterinaria y Agronomía, Universidad de Las Américas, Providencia, Chile; ^7^Laboratorio de Investigación en Agentes Antibacterianos, Departamento de Microbiología, Facultad de Ciencias Biológicas, Universidad de Concepción, Concepción, Chile

**Keywords:** antimicrobial resistance, *Escherichia coli*, dogs, drug resistance, genotypic, phenotypic

## Abstract

**Introduction:**

Antimicrobial resistance (AMR) is a major threat to animal and public health worldwide; consequently, several AMR surveillances programs have been implemented internationally in both human and veterinary medicine, including indicator bacteria such as *Escherichia coli*. However, companion animals are not typically included in these surveillance programs. Nevertheless, there have been reports of increasing levels of antimicrobial resistance in *E. coli* strains isolated from dogs worldwide. In Chile, there is limited information available on AMR in *E. coli* isolated from companion animals, which prevents the establishment of objective prevention and control measures.

**Methods:**

For this reason, the aim of this study was to characterize the phenotypic and genotypic AMR of *E. coli* strains isolated from healthy household dogs in Chile. For this purpose, a multi-stage sampling was carried out in the Metropolitan Region of Chile, obtaining samples from 600 healthy dogs. These samples were processed using traditional bacteriology and molecular techniques to isolate *E. coli* strains. We assessed the minimal inhibitory concentration of 17 antimicrobials and conducted a search of six antimicrobial resistance genes, as well as class 1 and 2 integrons, in the isolated strains.

**Results:**

Two-hundred and twenty-four strains of *E. coli* were recovered, and 96.9% (*n* = 217) showed resistance to at least one drug and only 3.1% (*n* = 7) were susceptible to all analyzed antimicrobials. Most strains were resistant to cefalexin (91.5%, *n* = 205, 1st-generation cephalosporin), followed by ampicillin (68.3%, *n* = 153) and cefpodoxime (31.3%, *n* = 70, 3rd-generation cephalosporin). Moreover, 24.1% (*n* = 54) tested positive for extended-spectrum-β-lactamases and 34.4% (*n* = 77) were multidrug resistant. As for the AMR genes, the most detected was *qnrB* (28.1%, *n* = 63), followed by *bla*_CTX-M_ (22.3%, *n* = 50), and *bla*_TEM-1_ (19.6%, *n* = 44). Additionally, 16.1% (*n* = 36) harbored class 1 integrons. Our study shows that *E. coli* strains isolated from healthy household dogs exhibit resistance to several relevant drugs and also antimicrobial resistance genes considered critical for human health. These results can be used as a starting point for the prevention and control of antimicrobial resistance from companion animals. This background should be considered when formulating future resistance surveillance programs or control plans in which companion animals must be included.

## 1. Introduction

Antimicrobial resistance (AMR) is defined as the ability of a microorganism to resist the growth inhibition or bactericidal activity of an antimicrobial, over the normal susceptibility of a specific bacterial species (1). Currently, one of the main threats to animal and public health, under the concept of “One Health,” is the emergence of resistant bacteria to several classes of antimicrobials (2).

The extent of the AMR phenomenon has led international organizations, such as the World Organization for Animal Health (WOAH), the Food and Agriculture Organization of the United Nations (FAO), the World Health Organization (WHO) and the United Nations Environment Programme (UNEP) to jointly promote the responsible use of antimicrobials in humans, animals and plants, under the concept of “One Health” (3, 4). Consequently, many countries have already implemented official plans for AMR control, including surveillance programs of bacteria isolated from healthy and diseased animals. However, only a few countries have implemented AMR surveillance schemes that include bacteria isolated from pets, such as the European Antimicrobial Resistance Surveillance Network (EARS-Net) or CEESA’s ComPath program (5). In general terms, these programs evaluate the susceptibility to different antibiotics in indicator bacteria, of which *Escherichia coli*, *Enterococcus faecium* and *E. faecalis* are the most used. These bacteria are part of the normal microbiota of different animal species, including dogs and humans, and have the ability to acquire new AMR genes from other bacteria and transfer them to other zoonotic or pathogenic bacteria by horizontal gene transfer (6–10).

According to WOAH data, companion animals receive almost 30% of the antimicrobials prescribed for animals, and many of these are the same as those used in human medicine, increasing the selection pressure for resistant bacterial strains (11). Thus, and according to various criteria, antimicrobials are classified into three categories according to WOAH: “critically important antimicrobials,” “highly important antimicrobials” and “important antimicrobials,” but they are oriented towards food-producing animals. The category of critically important veterinary antimicrobials includes fluoroquinolones, β-lactams, glycopeptides and polymyxins. These classes of antibiotics are also critically important for human medicine and are used in pets (12).

In Chile, the National Plan Against AMR has been implemented since 2017; however, to date no official AMR surveillance programs have yet been implemented in animals. Moreover, we have previously demonstrated that only a low percentage of veterinarians prescribe antibiotics with the support of microbiological diagnostic tools, and that the most frequently prescribed antimicrobials correspond to critical drugs, including penicillins, quinolones and cephalosporins (13), a situation that poses a threat for both animal and human health.

Considering the lack of information on AMR in bacteria isolated from companion animals in South America and Chile, coupled with the extensive use of critical drugs in those species, we aimed to characterize the phenotypic and genotypic AMR of *E. coli* strains isolated from healthy household dogs in the Metropolitan Region, Chile. In this way, we hope to reinforce current strategies that seek to increase awareness and regulation of the use of antimicrobials in dogs, as well as to generate updated scientific information that will allow the generation of therapeutic guidelines for the use of these drugs in companion animals.

## 2. Materials and methods

### 2.1. Study design

The design of this study was cross-sectional. Dogs were selected from veterinary clinics located in the Metropolitan Region (MR), which is Chile’s capital region. The sample size was calculated in 600 dogs using a sample size formula for estimating proportions proposed by Dohoo et al. (14), assuming a lack of knowledge about the proportion of dogs carrying antibiotic resistant *E. coli* (*a priori* proportion was set at 50%), and a precision of 4%, and a 95% confidence interval. Dogs were selected through a multi-stage sampling process with veterinary clinics as the primary sampling unit and dogs as the secondary unit. The sampling frame for veterinary clinics was built upon all businesses tagged with the term “clínica veterinaria” that were listed on Google Maps as of October 31, 2021; for each of them, the municipality and the macro-area were recorded. Between four and five veterinary clinics were selected from each of the seven MR macro-areas using a stratified random sampling scheme. Directors/owners of the selected veterinary clinics were contacted and invited to participate in the study. In the event of no response or a negative response to the invitation, another clinic was randomly selected from the same macro-area until the target sample size was reached. In each clinic, between 21 and 25 dogs that met the following conditions: (1) being healthy at the clinical examination, (2) not having been treated with antibiotics in the previous 4 weeks, and (3) residing in the same macro-area where the clinic was located, were selected in order of arrival.

### 2.2. Sample collection

Dogs were sampled with prior institutional (permit code 21439-VET-UCH) and signed owner consent in the MR. A total of 618 fecal samples belonging to the same number of dogs that attended 28 veterinary clinics during 2021–2022 were collected through rectal swabbing.

From dogs of any age and sex, clinically healthy, and without use of antibiotics during the 4 weeks prior to sampling, a swab with Cary Blair transport medium (Copan®, Murrieta, CA, USA) was inserted approximately 2 cm into the rectum and rotated gently for 10 s. After collection, all the samples were immediately refrigerated and transported to the laboratory within 4 h.

### 2.3. Sample processing

Once at the laboratory, samples were processed as previously described (15). Briefly, swabs were placed into 9 mL of trypticase soy broth (TS, BD®, Franklin Lakes, NJ, USA), homogenized in a vortex and incubated at 37°C for 18–24 h. After incubation, 50 μL were plated as screening on four MacConkey agar (BD®) plates for the detection of *E. coli*, which were incubated at 42°C for 18–24 h. Three of these plates were supplemented with a different antibiotic, including amoxicillin (16 μg/mL, Acros Organics®, Geel, Belgium), cefotaxime (2 μg/mL, Sigma®, San Luis, MO, USA), enrofloxacin (2 μg/mL, Sigma®), and one without antibiotics as a growth control. The selection of these drugs for plate supplementation was due to their critical relevance for both animal and human health and high reported use in companion animal clinical practice in Chile (12, 13). Strains growing on any of the antibiotic-supplemented plates were considered resistant, while those that did not grow on these plates but did grow on the control plates were considered susceptible. After incubation, one suspicious colony (red-pink colonies with bile precipitation) per plate was replated on a MacConkey agar plate without antibiotics.

### 2.4. Molecular identification of *Escherichia coli*

DNA from all suspicious colonies was extracted using the Wizard Genomic DNA purification kit (Promega, Madison, WI, USA), following the manufacturer’s instructions. Quality and concentration of DNA (260/280 absorbance ratio) was measured in a nanodrop (NANO-400 micro-spectrophotometer, Hangzhou Allsheng Instruments Co., Hangzhou, China). Samples with an absorbance ratio closest to the optimal range (1.8–2.0) were kept at –20°C for molecular analysis (16). *E. coli* identification was assessed by PCR on a LifeECO® thermal cycler (Hangzhou Allsheng Instruments Co.), according to the protocol described by Chen & Griffiths (17). Table 1 shows the sequence of primers used and the expected amplicon size. *E. coli* ATCC 25922 strain was used as positive control and *Salmonella* Typhimurium 14028 strain as negative control.

**Table 1:** Oligonucleotide sequences for *E. coli* identification, antimicrobial resistance genes and integrons, expected product size, and references.

Gene	Primers	Expected product size (bp)	References
*uspA*	F: CCGATACGCTGCCAATCAGT R: ACGCAGACCGTAGGCCAGAT	884	(17)
*uidA*	F: TATGGAATTTCGCCGATTTT R: TGTTTGCCTCCCTGCTGCGG	166	(17)
*qnrB*	F: GATCGTGAAAGCCAGAAAGG R: ACGATGCCTGGTAGTTGTCC	469	(18)
*qnrS*	F: ACGACATTCGTCAACTGCAA R: TAAATTGGCACCCTGTAGGC	417	(18)
*aac(6′)-Ib*	F: TTGCGATGCTCTATGAGTGGCTA R: CTCGAATGCCTGGCGTGTTT	482	(19)
*intI1*	F: CACGGATATGCGACAAAAAGGT R: ACATGGGTGTAAATCATCGTC	483	(20)
*intI2*	F: CACGGATATGCGACAAAAAGGT R: GTAGCAAACGAGTGACGAAATG	788	(20)
*bla*_CTX-M_	F: ATGTGCAGYACCAGTAARGTKATGGC R: TGGGTRAARTARGTSACCAGAAYCAGGG	593	(21)
*bla*_TEM-1_	F: ATCAGCAATAAACCAGC R: CCCCGAAGAACGTTTTC	445	(22)

### 2.5. Phenotypic antimicrobial resistance characterization

The minimal inhibitory concentration (MIC) of all confirmed *E. coli* strains was assessed using the automatized VITEK2 system (bioMérieux, Marcy-l’Étoile, France) to quantify its phenotypic AMR. This was carried out using the ASTGN98 card according to the manufacturer’s instructions, and clinical cut-off values according to the Clinical and Laboratory Standards Institute guidelines (23). The strains selected corresponded to all isolates growing on plates supplemented with cefotaxime and enrofloxacin. In the case of strains growing on plates supplemented with amoxicillin, they were only selected for MIC analysis if these strains also showed growth on any of the other plates supplemented with the other antibiotics (15). The ASTGN98 cards included aminoglycosides (amikacin and gentamicin), β-lactams (amoxicillin-clavulanic acid, ampicillin, cefalexin, cefovecin, cefpodoxime, ceftazidime, ceftiofur, and imipenem), folate synthesis inhibitors (trimethoprim-sulfamethoxazole), nitrofurans (nitrofurantoin), phenicols (chloramphenicol), quinolones (ciprofloxacin, enrofloxacin, and marbofloxacin), tetracyclines (doxycycline). For the detection of extended-spectrum β-lactamases (ESBL) these cards also include cefepime, cefotaxime, ceftazidime alone, and in combination with clavulanic acid. Strains were classified as susceptible, intermediate susceptibility or resistant according to the MIC values (24). Multidrug resistance (MDR) was confirmed when a strain presented resistance to three or more antibiotics of different classes (25). Additionally, the multiple antimicrobial resistance (MAR) index was calculated as “a/b,” where “a” corresponds to the number of antimicrobials for which a particular isolate was resistant and “b” the total number of antimicrobials tested (26).

### 2.6. Genotypic antimicrobial resistance characterization

The presence of six AMR genes in all *E. coli* strains was assessed by PCR in a LifeECO Thermocycler (Hangzhou Allsheng Instruments Co.) with previously obtained DNA. The genes analyzed included *bla*_TEM − 1_ and *bla*_CTX − M_ for β-lactamases; *qnrB*, *qnrS* and *aac(6′)-Ib-cr* for plasmid-mediated resistance to quinolones; and *aac(6′)-Ib* for aminoglycosides-modifying enzymes. Additionally, the presence of class 1 and class 2 integrons was also assessed by detecting the *intI1* and *intI2* genes. These genes were selected given their reported distribution in *E. coli* strains and because they encode resistance to critically important drugs for human and veterinary medicine, posing a risk to animal and public health (27, 28). All PCR reactions were performed in duplicate.

Strains belonging to our collection, whose PCR products for the detection of the aforementioned genes were sequenced and their nucleotide identity corroborated by comparison to sequences deposited at GenBank (National Center for Biotechnology Information, Bethesda, MD, USA) (data not published), were used as positive controls. Table 1 summarizes all primers used for molecular detection of AMR genes. All PCR products positive for *aac(6′)-Ib* were further analyzed by digestion with BtsCI (NewEngland Biolabs, Ipswich, MA, USA) to identify the *aac(6′)-Ib-cr* allele, which lacks the BtsCI restriction site present in the wild-type gene (29, 30). All digestion products were sequenced and compared to the database available in GenBank® (National Center for Biotechnology Information, Bethesda, MD, USA) to establish their nucleotide identity (NI), which was confirmed considering ≥97% of identity.

### 2.7. Statistical analyses

The phenotypic and genotypic resistance patterns were studied through multiple correspondence analysis (MCA). In the case of phenotypic resistance, MCA was used to evaluate the proximal relationships of the resistant/susceptible status to the different antibiotics among *E. coli* isolates. For the genotypic resistance analysis, MCA was aimed to assess the relationships of the presence/absence of AMR genes among the isolates. In all cases, MCAs were limited to the derivation of two dimensions. The relationships among the antibiotics’ resistant/susceptible condition, and among the presence/absence of resistance genes were plotted by means of two-dimensional correspondence maps. All MCAs were carried out in Stata v15 (StataCorp, College Station, TX, USA).

## 3. Results

### 3.1. Isolation of resistant *Escherichia coli* isolates at plate screening

From all samples, 489 (79.1%) *E. coli* isolates were recovered after plating onto any of the supplemented plates. From these isolates, 34.1% (*n* = 211) were obtained on amoxicillin-supplemented plates; 9.2% (*n* = 57) on plates with cefotaxime; and 15.7% (*n* = 97) on plates with enrofloxacin. As some samples grew on more than one supplemented plate, 224 isolates were selected.

### 3.2. Phenotypic antimicrobial susceptibility characterization of *Escherichia coli* isolates

After selection of all *E. coli* isolates, 224 were characterized on their MIC. Of the total strains, 96.9% (*n* = 217) showed resistance to at least one drug and only 3.1% (*n* = 7) were susceptible to all analyzed antimicrobials. Thus, most of them were resistant to cefalexin (91.5%, *n* = 205), followed by ampicillin (68.3%, *n* = 153) and cefpodoxime (31.3%, *n* = 70). On the other hand, no strains were resistant to imipenem or nitrofurantoin. Additionally, 24.1% (*n* = 54) were positive for ESBL and 34.4% (*n* = 77) were MDR. Table 2 shows the detection rates of resistant strains and the MIC_50_ and MIC_90_ for each analyzed antibiotic.

**Table 2:** Isolation rates and MICs of *E. coli* strains isolated from dogs for each of the antimicrobials analyzed.

Antimicrobial class	Antimicrobial	Number of resistant strains (%)	MIC_50_ (μg/mL)	MIC_90_ (μg/mL)	Range (μg/mL)	Breakpoint		
Aminoglycosides	AMK	1 (0.4)				S	I	R
	GEN	15 (6.7)	≤2	≤2	≤2–16	≤4	8	≥16
β-lactams	AMC	41 (18.3)	≤1	≤1	≤1- ≥ 16	≤2	4	≥8
	AMP	153 (68.3)	8	16	≤2- ≥ 32	≤8	16	≥32
	LEX	205 (91.5)	≥32	≥32	≤2- ≥ 32	≤8	-	≥16
	CFO	68 (30.4)	8	≥64	≤4- ≥ 64	≤2	4	≥8
	CPD	70 (31.3)	≤0.25	≤8	≤0.5- ≥ 8	≤2	4	≥8
	CAZ	15 (6.7)	≤0.25	≥8	≤0.25- ≥ 8	≤2	4	≥8
	CFT	66 (29.5)	≤0.12	8	≤0.12- ≥ 64	≤4	8	≥16
	IPM	0	≤1	≥8	≤1- ≥ 8	≤2	4	≥8
Folate synthesis inhibitors	SXT	57 (25.4)	≤0.25	≤0.25	≤0.25–0.5	≤1	2	≥4
Nitrofurans	NIT	0	≤20	≥320	≤20- ≥ 320	≤38	-	≥76
Phenicols	CHL	54 (24.1)	≤16	≤16	≤16–64	≤32	64	≥128
Quinolones	CIP	55 (24.6)	≤8	≥64	≤2- ≥ 64	≤8	16	≥32
	ENR	55 (24.6)	0.5	≥4	≤0.06- ≥ 4	≤1	2	≥4
	MRB	53 (23.7)	1	≥4	≤0.12- ≥ 4	≤0,5	1	≥4
Tetracyclines	DOX	69 (30.8)	≤0.5	≥4	≤0.5- ≥ 4	≤1	2	≥4
			≤1	≥16	≤0.5- ≥ 16	≤4	8	≥16

AMK, Amikacin; GEN, gentamicin; AMC, amoxicillin-clavulanic acid; AMP, ampicillin; LEX, cefalexin; CFO, cefovecin; CPD, cefpodoxime; CAZ, ceftazidime; CFT, ceftiofur; IPM, imipenem; SXT, trimethoprim-sulfamethoxazole; NIT, nitrofurantoin; CHL, chloramphenicol; CIP, ciprofloxacin; ENR, enrofloxacin; MRB, marbofloxacin; DOX, doxycycline; S, susceptible; I, intermediate resistant; R, resistant. Breakpoints were obtained from CLSI (23).

Among resistant strains 72 resistance profiles were detected, with cefalexin alone (20.1%, *n* = 45), ampicillin-cefalexin (5.8%, *n* = 13), and ampicillin-cefalexin-cefpodoxime-cefovecin-ceftiofur and ampicillin-cefalexin-cefpodoxime-cefovecin-ceftiofur-ciprofloxacin-enrofloxacin-marbofloxacin-trimethoprim-sulfamethoxazole (4.9%, *n* = 11 each) the most frequently detected. Supplementary Table S1 shows the antimicrobial resistance patterns of all the analyzed isolates.

Regarding the MAR index, the highest value detected was 0.8, corresponding to a strain showing resistance against ampicillin, amoxicillin-clavulanic acid, cefalexin, cefpodoxime, cefovecin, ceftiofur, gentamicin, ciprofloxacin, enrofloxacin, marbofloxacin, doxycycline, chloramphenicol, and trimethoprim-sulfamethoxazole.

### 3.3. Genotypic antimicrobial resistance characterization of *Escherichia coli* isolates

As for the AMR genes, the most detected was *qnrB* (28.1%, *n* = 63), followed by *bla*_CTX-M_ (22.3%, *n* = 50), *bla*_TEM-1_ (19.6%, *n* = 44), *qnrS* (11.6%, *n* = 26) and *aac(6’)Ib-cr* (1.3%, *n* = 3 each). Additionally, 16.1% (*n* = 36) harbored class 1 integrons, while the gene *aac(6’)Ib* and class 2 integrons were not detected. Twenty-four profiles of AMR genes were detected, were *qnrB* alone was the most frequent (20.1%, *n* = 45), followed by *bla*_CTX-M_ alone (11.6%, *n* = 26), *bla*_TEM-1_ alone (5.8%, *n* = 13) and *bla*_TEM-1_*-qnrB* (4.5%, *n* = 10). All gene profiles are included in Supplementary Table S1.

### 3.4. Multiple correspondence analysis

**Figure 1:**
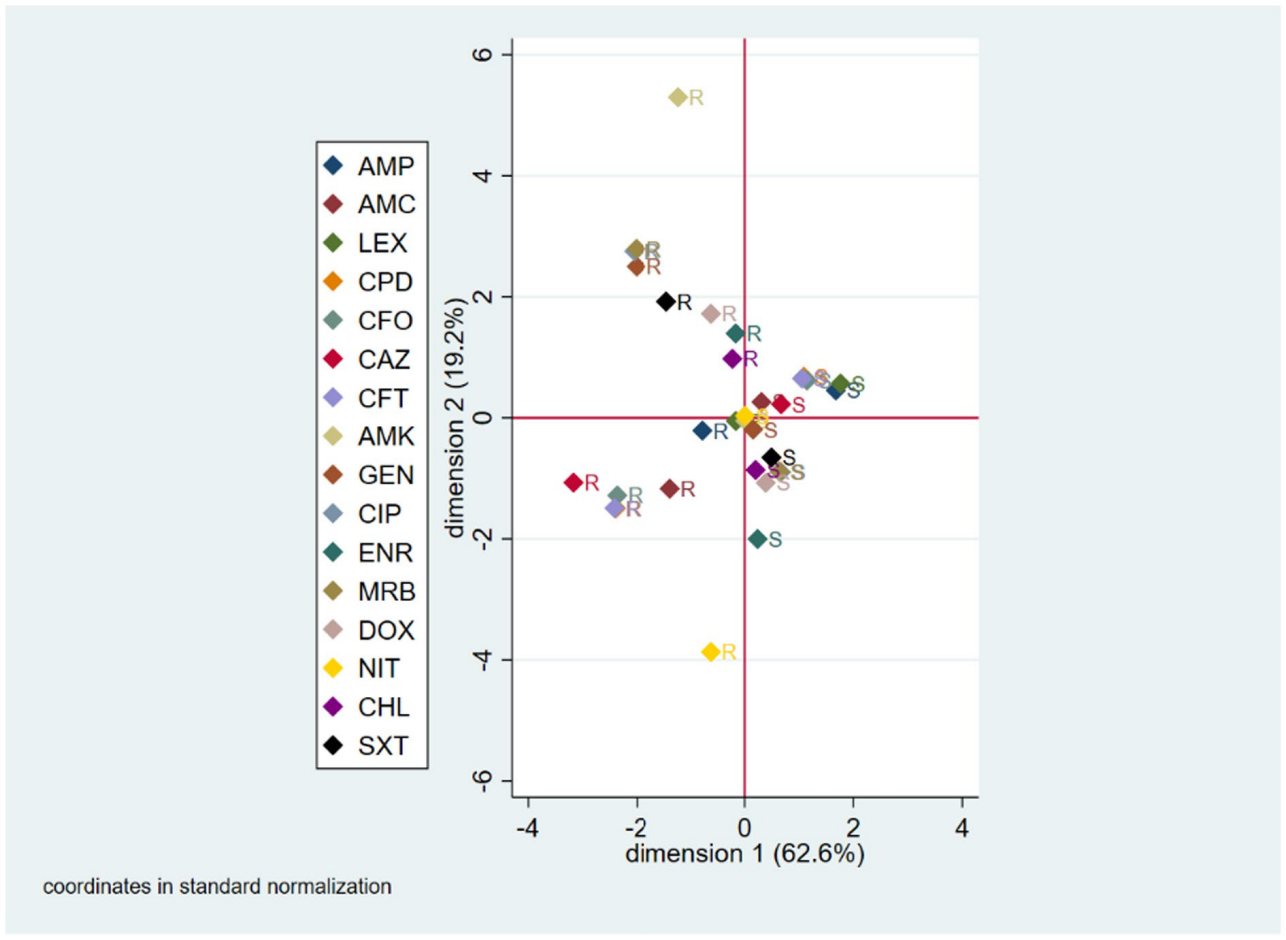
Multiple correspondence analysis (MCA) coordinate plot for phenotypic AMR characterization. S, susceptible; R, resistant; AMK, amikacin; AMC, amoxicillin-clavulanic acid; AMP, ampicillin; LEX, cefalexin; CFO, cefovecin; CPD, cefpodoxime; CAZ, ceftazidime; CFT, ceftiofur; CHL, chloramphenicol; CIP, ciprofloxacin; DOX, doxycycline; ENR, enrofloxacin; GEN, gentamicin; IPM, imipenem; MRB, marbofloxacin; NIT, nitrofurantoin; SXT, trimethoprim-sulfamethoxazole.

Multiple correspondence analysis (MCA) for the phenotypic analysis included all tested antibiotics, except imipenem since all *E. coli* isolates were susceptible to it. The first and second derived dimensions together accounted for 81.8% of the total variability of the resistant/susceptible status among antibiotics (first dimension = 62.6%; second dimension = 19.2%). The two-dimensional correspondence plot shows that the resistance/susceptibility status of the isolates was grouped according to the different antibiotics. Isolates sensitive to trimethoprim-sulfamethoxazole generally were also susceptible to chloramphenicol, doxycycline, marbofloxacin, ciprofloxacin and to a lesser extent to enrofloxacin; therefore, the isolates resistant to any of these drugs, generally, were also resistant to the others. An equivalent situation was observed with isolates susceptible/resistant to cefpodoxime, ceftiofur, cefovecin, and to a lesser extent to cefalexin and ampicillin. The plot also suggests that when an isolate was susceptible to amikacin, it was also susceptible to gentamicin, amoxicillin-clavulanic acid, and ceftazidime, but resistant to cefalexin and ampicillin (Figure 1).

**Figure 2:**
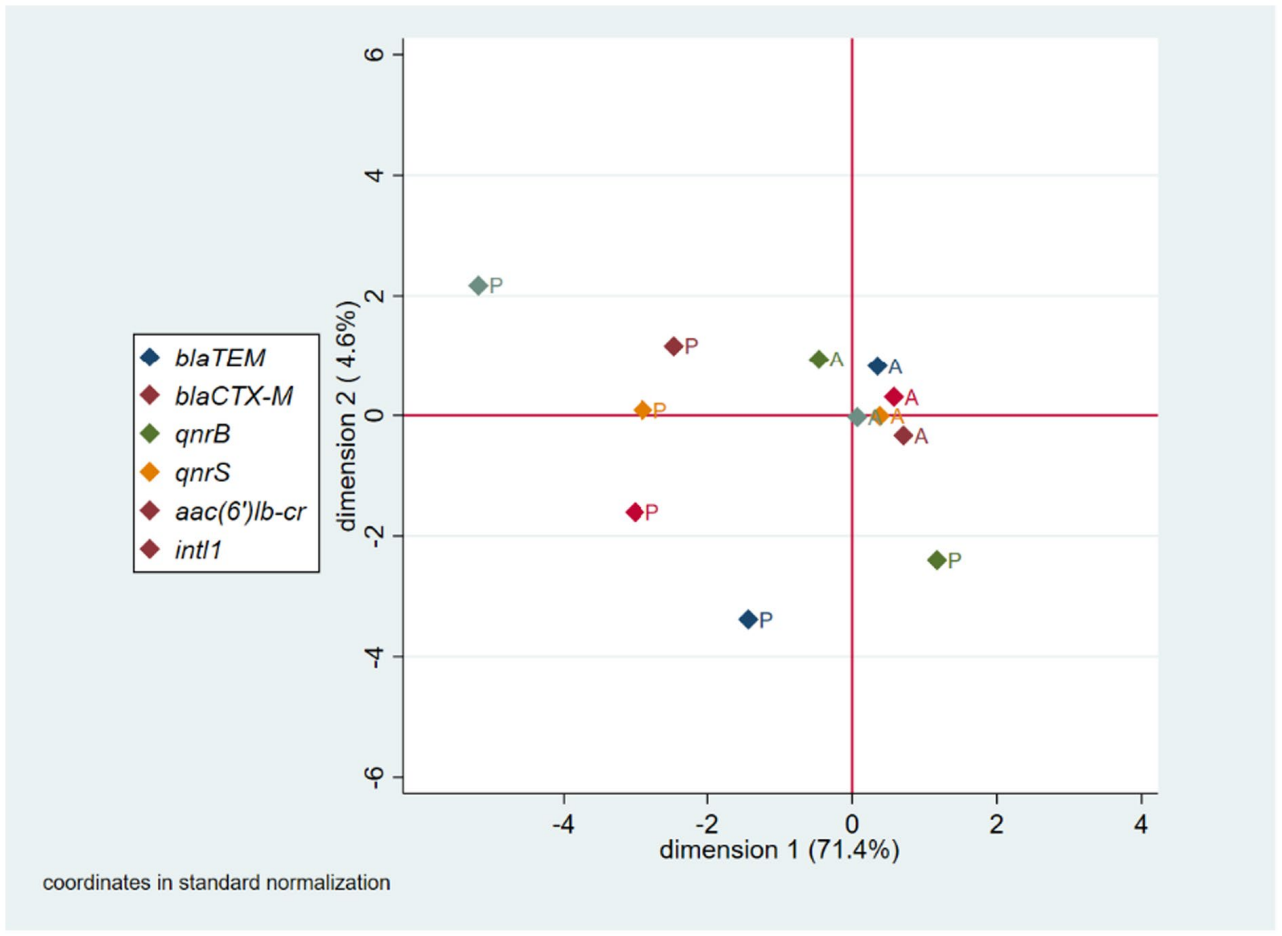
MCA coordinate plot for genotypic AMR characterization. A, absence; P, presence.

The MCA performed to evaluate proximal relationships for the presence/absence of genes included *bla*_TEM_, *bla*_CTX-M_, *qnrB*, *qnrS*, *aac(6’)Ib-cr*, and *intI1* genes, as these were both present and absent across isolates. The resulting two-dimension model accounted for 76.0% of the total variance of the original variables (first dimension = 71.4%; second dimension = 4.6%). The MCA two-coordinate plot shows a clustering of the gene absence condition, which means that the isolates mainly agree in the absence of genes, rather than in the presence. Those pairs of genes in which this relationship was seen to be more marked were *qnrS* and *aac(6’)Ib-cr*, as they both were absent in 88% of the isolates. The percentage of gene absence coincidence for the *qnrS*-*intI1*, *bla*_TEM_-*intI1*, and *bla*_CTX-M_-*intI1* pairs were 76, 71, and 69%, respectively. The gene pair with the highest percentage of coincidence in the presence of genes was *qnrS*-*bla*_CTX-M_ with only 5% of isolates (Figure 2).

## 4. Discussion

Use of antimicrobials (inappropriate or appropriate), inadequate or nonexistent programs for infection prevention and control, poor-quality medications, weak laboratory capacity, inadequate surveillance, and insufficient regulation for the use of antimicrobials are crucial factors that contribute to the worldwide spread of AMR (3). Most studies have focused on strains isolated from humans, and less attention has been given to strains isolated from companion animals, minimizing their role as potential reservoirs and disseminators of those strains to other animals, their owners, and the environment.

Antimicrobial-resistant *E. coli* strains isolated from companion animals have been detected worldwide and represent one of the greatest challenges to public health, especially MDR, fluoroquinolone-resistant and ESBL-producing strains (31–34). However, studies in Latin America on this subject are still scarce (34–36), pointing to the risk to animal and public health that these strains pose, since the resistance patterns are country specific.

Although in Chile there are no official AMR surveillance plans for *E. coli* strains isolated from animals; to date, there are only two published articles describing AMR of *E. coli* isolated from dogs. In the first, Moreno et al. (37) investigated the presence of ESBL among third-generation cephalosporin resistant strains of *E. coli* isolated from feces obtained from dogs treated and not treated with enrofloxacin. Among the 52 strains isolated from enrofloxacin-treated animals the resistance to third-generation cephalosporins ranged between 23% (cefotaxime, ceftazidime) and 27% (cefpodoxime); while the AMR to enrofloxacin was 86.5%, followed by ciprofloxacin (82.7%), levofloxacin (80.8%) and moxifloxacin (80.8%). On the other hand, among the 18 isolates from the control group, no resistant strains to third-generation cephalosporins or to fluoroquinolones were detected. Additionally, in 14 strains isolated from treated dogs ESBL were detected, while none was detected in the control group. Among those 14 strains, 50% harbored the genes *bla*_CTX-M-1_, *bla*_TEM_ and *bla*_PER-2_; 35.7% *bla*_CTX-M-14_ and *bla*_TEM_; 7.1% *bla*_CTX-M-1_ and *bla*_TEM_; while 7.1% possessed *bla*_CTX-M-14_, *bla*_TEM_ and *bla*_PER-2_. In the second study, Benavides et al. (38) investigated the prevalence of ESBL-producing *E. coli* strains in 82 dogs belonging to agricultural settings in Central Chile. Thus, they detected a prevalence of 24.4% and isolating 32 strains of ESBL-producing *E. coli*; of which more than 20% were resistant to ciprofloxacin, chloramphenicol, sulfamethoxazole, and tetracycline; with 47% being MDR. Among those strains, 78% carried *bla*_CTX-M-1_, 63% *bla*_CTX-M-2_, 12.5% *bla*_CTX-M-9_, 3% *bla*_CTX-M-8_ and 3% *bla*_CTX-M-25_. Still, to the best of our knowledge, ours is the first published study investigating the AMR of *E. coli* strains isolated from household dogs against critical drugs and with a region-wide approximation.

Regarding phenotypic AMR in the *E. coli* strains analyzed, our results show that resistance against β-lactams was the most frequent, followed by tetracyclines, folate synthesis inhibitors, fluoroquinolones, chloramphenicol, and aminoglycosides. Furthermore, our MCA for phenotypic AMR characterization suggests, as expected, the presence of simultaneous resistance to drugs from the same family, such as ciprofloxacin and marbofloxacin, or third generation cephalosporins such as ceftiofur, cefpodoxime and cefovecin; and the association of resistance to drugs from different families, such as enrofloxacin and chloramphenicol. On the other hand, the MCA for genotypic AMR suggests the absence of clusters of resistant strains. In other words, when one of these genes is present in an isolate, the others are unlikely to be present as well. This situation may be due to the high clonal diversity of the isolated strains and probably to the absence of mobile genetic elements harboring several resistance genes simultaneously. However, more detailed studies are needed to confirm these findings, and we hope to publish these results in the near future.

Among β-lactams, in human medicine 3rd and 4th generation cephalosporins, carbapenems, antipseudomonal penicillins, and aminopenicillins with and without β-lactamase inhibitors are considered of critical importance; and 1st and 2nd generation cephalosporins, amidinopenicillins, anti-staphylococcal, and narrow spectrum penicillins of highly importance. In a similar way, in veterinary medicine 3rd and 4th generation cephalosporins, penicillins are considered of critical importance; and 1st and 2nd generation cephalosporins of highly importance (39, 40). However, β-lactam resistance in *E. coli* strains isolated from dogs is well-documented internationally, a scenario that has been complicated by the rapid distribution of ESBL-producing strains. In this context, Thungrat et al. (41) reported similar levels of AMR in 3,172 *E. coli* strains isolated from dogs in the United States. Among those strains, 98% were resistant to cefalotin, 52.7% to ampicillin, 45.2% to amoxicillin-clavulanic acid, 19.5% to ticarcillin-clavulanic acid, 13.9% to cefpodoxime, 8.7% to cefoxitin, 10.1% to ceftazidime, and 10.9% to cefotaxime. Moreover, the MIC_90_ for some drugs was higher than ours, namely ampicillin (>256 μg/mL), amoxicillin-clavulanic acid (32 μg/mL), cefalotin (compared with cephalexin, 512 μg/mL). More recently, Chen et al. (33) analyzed the AMR of 127 *E. coli* strains isolated from clinical samples from dogs in China between 2012 and 2017, registering 78% of resistance to ampicillin, 65.4% to cefazolin, 58.3% to cefotaxime and ceftriaxone and 22.8% to amoxicillin-clavulanic acid. Also, they demonstrated a significant increase in AMR rates from 2012 to 2017, with rates to cefotaxime, ceftriaxone, cefazolin, and amoxicillin + clavulanic acid rates more than doubled. Regarding the detection of ESBL-producing *E. coli* strains isolated from dogs, Salgado-Caxito (42) conducted a meta-analysis revealing a global prevalence of 6.87% (95% CI, 4.46–10.45%), rate lower than our findings (24.1%), which represents a serious threat to public health. The phenotypic resistance against β-lactams could be provided by several resistance genes encoding β-lactamases, including those belonging to the TEM, SHV and CTX-M families, among others (43). In our study, β-lactams resistance genes were detected at lower rates (*bla*_CTX-M_ 22.3%, *bla*_TEM-1_ 19.6%) than the phenotypic resistance. In fact, here 125 out of the 205 *E. coli* strains resistant to cefalexin (60.9%) did not harbor *bla*_TEM-1_ or *bla*_CTX-M_ genes, as did 79 out of the 153 strains resistant to ampicillin (51.6%) and 31 out of the 41 strains resistant to amoxicillin-clavulanic acid (75.6%). These discrepancies may be due to the presence of other β-lactamase encoding genes, such as *bla*_TEM-2_, *bla*_TEM-10_, plasmid-encoded AmpC or SHV and PER enzymes (43); or even by the deficiencies in outer membrane porins, such as OmpC and OmpF (44, 45). Conversely, 88.9% (48/54) of the ESBL-producing strains harbored *bla*_CTX-M_, being all of them resistant to ampicillin and cefalexin, 98.1% to cefpodoxime, 96.3% to cefovecin, 90.7% to ceftiofur, 12.9% to amoxicillin-clavulanic acid and 9.3% to ceftazidime. These results were expected, as most CTX-M enzymes are inhibited by clavulanic acid and do not efficiently hydrolyze ceftazidime (46). Our results are of great concern as ESBL-producing *Enterobacteriaceae* represent a serious threat for both veterinary and human medicine and hence, are considered of high importance for the research, discovery and development of new antimicrobials to face these infections (47).

Tetracyclines belong to a family of broad-spectrum antibiotics, where its efficacy, low cost, and the lack of side effects make them widely used in animals. In pets, doxycycline is an antibiotic of choice for different diseases; therefore, its use is common. In Chile, doxycycline has been reported to be the fourth most widely used antimicrobial in companion animals (13). Such widespread use of tetracycline antibiotics has resulted in selection of resistance in indicator bacteria, and its intensive use in veterinary medicine has caused a high prevalence of tetracycline resistance (48). Tetracycline resistance occurs most often as a result of the acquisition of *tet* genes that code for efflux pumps or ribosomal protection (49). Efflux resistance genes are generally found on plasmids, whereas genes involved in ribosomal protection have been found on both plasmids and conjugative transposons, highlighting the transference potential of these genes to other bacteria, environments, animals, and humans (50). In this context, Wedley et al. (51) reported 24.5% of tetracycline resistance in *E. coli* strains isolated from 183 healthy dogs from a semi-rural community in England, with 12 of those strains harboring the *tet(B)* gene. In our study, resistance against tetracyclines was the second highest detected, with 30.8% of resistance to doxycycline and a high MIC_90_ (>16 μg/mL), but lower than expected due to the wide use of this family of drugs in the clinical practice of companion animals in Chile. However, the presence of *tet* genes was not investigated, which will be included in a subsequent whole genome sequencing study of these strains.

One antimicrobial family with phenotypic resistance percentages close to 25% was the folate synthesis inhibitors. Sulfonamides and trimethoprim interfere with the formation of folic acid in bacteria, thus exerting its bactericidal effect. Since these drugs attack consecutive steps in the same enzymatic pathway, they have a synergistic effect, which has been successfully exploited in a broad-spectrum combination drug, cotrimoxazole (52). Nevertheless, resistance to both agents has developed rapidly among all major species of bacteria (53). In *E. coli*, this resistance may be due to chromosomal mutations of *folP* or *folA* genes, or by the plasmid-borne *sul* and *dhfr* genes (52). Susceptibility testing is usually performed for the sulfonamides and trimethoprim combination; however, results of tests performed on either drug alone are scarce. Here, we registered 25.4% of resistance against trimethoprim-sulfamethoxazole, with a high CIM_90_ of ≥320 μg/mL, a rate slightly higher than internationally registered data from *E. coli* strains isolated from dogs, with values ranging from 5.1–19.1% (34, 36, 41, 54). Conversely, are higher than the reported use of these drugs in the companion animals’ practice in Chile (13).

Fluoroquinolones are antimicrobials widely used in human and veterinary medicine, and due to their therapeutic relevance are considered critically important by both WHOA and WHO (39, 40). However, these drugs are so widely prescribed that they have led to the development of a great selective pressure for the emergence of strains with reduced susceptibility to them. Such strains have appeared among nearly all species against which fluoroquinolones have activity (55). The target sites of action in *E. coli* strains are bacterial topoisomerases, DNA gyrase (topoisomerase II) as a primary site and topoisomerase IV as a secondary target. Mutations in specific domains of *gyrA*, *gyrB*, *parC*, and *parE* cause changes in gyrase or topoisomerase IV that contribute to quinolone resistance, which can be transmitted vertically. In addition, to date, three families of plasmid-mediated mechanisms associated with quinolone resistance have been identified: Qnr proteins that protect the DNA gyrase and topoisomerase IV from inhibition by quinolones; aminoglycoside acetyltransferase variant *aac(6′)-Ib-cr* that acetylates ciprofloxacin and norfloxacin; and efflux pumps QepA and OqxAB that remove antibiotics from bacterial cells (56). Here, we registered an overall resistance to fluoroquinolones near 25%, with a MIC_90_ of >4 μg/mL. Of these strains, 53 were resistant to ciprofloxacin, enrofloxacin and marbofloxacin; one was resistant only to ciprofloxacin; other resistant only to enrofloxacin; and other one resistant to both ciprofloxacin and enrofloxacin. Moreover, 34.4% of all strains showed intermediate resistance to enrofloxacin and 0.4% to marbofloxacin. These phenotypic resistance detection rates are higher than those reported by Moreno et al. (37), where no fluoroquinolones-resistant *E. coli* strains were detected from healthy dogs not treated with antimicrobials. This could indicate that the widespread use of this family of antimicrobials has generated sufficient selection pressure for healthy animals without antimicrobial use to carry fluoroquinolone-resistant strains in their gut. In fact, according to Galarce et al. (13), quinolones are reported as the second most used therapeutic alternative in the clinical practice of companion animals in Chile. Additionally, we also detected the presence of the plasmid-borne fluoroquinolone resistance genes *qnrB*, *qnrS* and *aac(6’)Ib-cr*. Noteworthy, only 10 fluoroquinolone-resistant strains harbored *qnrB* and only five *qnrS*, being mainly detected in susceptible strains. In the case of the three strains harboring the *aac(6’)Ib-cr*, all of them were resistant to fluoroquinolones, but one also carried the *qnrB* and *qnrS* genes, and two the *bla_CTX-M_* gene. This is not surprising because, plasmids harboring fluoroquinolone resistant genes often carry other antibiotic resistance genes conferring resistance to β-lactams, aminoglycosides, chloramphenicol, tetracycline, sulfonamides, trimethoprim, and rifampin, allowing the co-selection of MDR strains (57). These results confirm that *E. coli* isolates from healthy dogs can be an important source of spread of critical antibiotic resistance determinants.

Bacterial resistance to phenicols is widely reported worldwide, mainly in production animals. However, the detection of this resistance in bacteria isolated from companion animals is striking, as its use is rather restricted. In fact, in Chile according to official data the sale of this family of antimicrobials for use in non-productive animals for the year 2021 was nil (58); and the reported use of these molecules in companion animals represents only 0.3% (13). This resistance is mainly due to the presence of acetyltransferases encoded by *cat* genes that inactivate chloramphenicol, which is frequently located within mobile elements such as plasmids, transposons, or gene cassettes and are able to be transferred between bacteria of different species (49). Previously in Chile, Moreno et al. (37) registered 11.5% of florfenicol resistance in *E. coli* strains isolated from enrofloxacin-treated dogs. Here, we detected 24.1% of AMR to chloramphenicol, which could indicate an increase in this resistance in the national dog population; and also, is not consistent with the use of these molecules in dogs but could be explained by the dissemination of their genetic resistance determinants in *E. coli* strains present in dogs.

In addition to the above, our study indicates that 34.4% of the strains analyzed were resistant to three or more antibiotic families (MDR). Among the resistant strains, 72 resistance profiles were detected; and of these, six strains were resistant to 10 antibiotics simultaneously; one strain showed resistance to 11 antibiotics simultaneously; three strains to 12 antibiotics; and one strain to 13 antibiotics. Regarding the presence of integrons, only class 1 integrons were detected in 16.1% of the analyzed strains, and of them 69.4% showed MDR. Integrons are natural recombination and expression systems with the ability to acquire gene cassettes; and although they are not mobile elements, they are frequently associated with mobile elements such as conjugative plasmids, insertion sequences and transposons, making them one of the most important elements in the dissemination of resistance (59). More than 70 different gene cassettes conferring resistance to most of the known β-lactams, aminoglycosides, trimethoprim, rifampicin, chloramphenicol, quinolones, erythromycin and quaternary ammonium compounds have been reported in this class of integron (60). This may indicate that the MDR registered here is possibly transmitted horizontally to other bacteria or to the environment, where the close contact between companion animals and people as well as the shared home environment facilitate the transmission of this antibiotic-resistant bacteria and/or their AMR encoding genes (61).

Although current legislation in Chile establishes that antimicrobials should only be used for therapeutic or metaphylactic purposes; that fluoroquinolones and third and fourth generation cephalosporins should not be used as a first line of treatment, except when there are no effective alternatives; that the use of fluoroquinolones and third and fourth generation cephalosporins as a second therapeutic alternative should be supported by bacterial susceptibility studies; and that all antimicrobials should be sold with a veterinary prescription (62), our results show that these measures have been ineffective in reducing the spread of AMR, including that against critical drugs. This situation points to the need to establish integrated surveillance programs under the One Health concept to determine the epidemiological status of AMR at the human-animal-environment interface; to standardize detection protocols at the national level; and to strengthen awareness of prescribers, pet keepers and the community.

Finally, our study shows that *E. coli* strains isolated from healthy dogs exhibit resistance to several relevant drugs and also antimicrobial resistance genes considered critical for human health. As observed, healthy dogs have *E. coli* strains resistant to third-generation cephalosporins and fluoroquinolones, and many of them are resistant to both families of antimicrobials. These results can be used as a starting point for the prevention and control of antimicrobial resistance in companion animals. This background should be considered when formulating future resistance surveillance programs or control plans in which companion animals must be included.

## Data availability statement

The original contributions presented in the study are included in the article/Supplementary material, further inquiries can be directed to the corresponding authors.

## Ethics statement

The animal studies were approved by Comité Institucional de Cuidado y Uso de Animales of the Universidad de Chile (permit code 21439-VET-UCH). The studies were conducted in accordance with the local legislation and institutional requirements. Written informed consent was obtained from the owners for the participation of their animals in this study.

## Author contributions

NG, GA, and LL contributed to the conception and design of the study and wrote the first draft of the manuscript. NG, GA, LL, EP-O, and GG-R contributed with resources to the study. FS, BE, MM, SM, RV, CV, JP, and CZ performed the laboratory analyses. GA performed the statistical analysis. All authors contributed to the article and approved the submitted version.

## Funding

This work was supported by the Fondo Nacional de Desarrollo Científico y Tecnológico (FONDECYT) grant number 1210692.

## Conflict of interest

The authors declare that the research was conducted in the absence of any commercial or financial relationships that could be construed as a potential conflict of interest.

## Publisher’s note

All claims expressed in this article are solely those of the authors and do not necessarily represent those of their affiliated organizations, or those of the publisher, the editors and the reviewers. Any product that may be evaluated in this article, or claim that may be made by its manufacturer, is not guaranteed or endorsed by the publisher.
